# Interpretable Prediction of Myocardial Infarction Using Explainable Boosting Machines: A Biomarker-Based Machine Learning Approach

**DOI:** 10.3390/diagnostics15172219

**Published:** 2025-09-01

**Authors:** Zeynep Kucukakcali, Ipek Balikci Cicek, Sami Akbulut

**Affiliations:** 1 Department of Biostatistics and Medical Informatics, Inonu University Faculty of Medicine, Malatya 44280, Turkey; 2 Department of Surgery, Inonu University Faculty of Medicine, Malatya 44280, Turkey

**Keywords:** myocardial infarction, biomarkers, machine learning, explainable artificial intelligence, explainable boosting machine, clinical decision support

## Abstract

**Background/Objectives**: This study aims to build an interpretable and accurate predictive model for myocardial infarction (MI) using Explainable Boosting Machines (EBM), a state-of-the-art Explainable Artificial Intelligence (XAI) technique. The objective is to identify and rank clinically relevant biomarkers that contribute to MI diagnosis while maintaining transparency to support clinical decision making. **Methods**: The dataset comprises 1319 patient records collected in 2018 from a cardiology center in the Erbil region of Iraq. Each record includes eight routinely measured clinical and biochemical features, such as troponin, CK-MB, and glucose levels, and a binary outcome variable indicating the presence or absence of MI. After preprocessing (e.g., one-hot encoding, normalization), the EBM model was trained using 80% of the data and tested on the remaining 20%. Model performance was evaluated using standard metrics including AUC, accuracy, sensitivity, specificity, F1 score, and Matthews correlation coefficient. Feature importance was assessed to identify key predictors. Partial dependence analyses provided insights into how each variable affected model predictions. **Results**: The EBM model demonstrated excellent diagnostic performance, achieving an AUC of 0.980, an accuracy of 96.6%, sensitivity of 96.8%, and specificity of 96.2%. Troponin and CK-MB were identified as the top predictors, confirming their established clinical relevance in MI diagnosis. In contrast, demographic and hemodynamic variables such as age and blood pressure contributed minimally. Partial dependence plots revealed non-linear effects of key biomarkers. Local explanation plots demonstrated the model’s ability to make confident, interpretable predictions for both positive and negative cases. **Conclusions**: The findings highlight the potential of EBM as a clinically useful and ethical AI approach for MI diagnosis. By combining high predictive accuracy with transparency, EBM supports biomarker prioritization and clinical risk stratification, thus aligning with precision medicine and responsible AI principles. Future research should validate the model on multi-center datasets and explore additional features for broader clinical use.

## 1. Introduction

Cardiovascular diseases, particularly myocardial infarctions (MIs), remain a major global health challenge due to their substantial contribution to morbidity and mortality [1,2]. In 2019, cardiovascular diseases accounted for approximately 32% of all deaths worldwide, with heart attacks alone responsible for nearly 17 million annual deaths, as reported by the World Health Organization (WHO) [3]. Acute MI not only poses an immediate life-threatening condition but also results in long-term health complications, including the development of heart failure and recurrent cardiovascular events [4,5,6].

Epidemiological trends over the past two decades have illustrated a nuanced shift in the global burden of MI. In several high-income countries, a gradual decline in MI incidence has been observed, primarily due to advances in acute cardiac care, public health, and preventive strategies [7,8]. However, this overall improvement conceals persistent disparities. Demographic and socioeconomic inequalities continue to influence MI outcomes, with disadvantaged populations experiencing disproportionately higher risks and less favorable trends over time [7,9]. The observed inequities in cardiovascular outcomes are closely linked to systemic differences in healthcare access, patient education regarding cardiovascular risk factors, and delays in symptom recognition and treatment initiation. As Kristono [10] highlights, populations with lower socioeconomic status often face structural barriers that hinder timely medical intervention, including limited access to smoking cessation programs and cardiovascular medications.

Despite some progress in reducing incidence, mortality following heart attacks remains a serious concern. Many deaths occur shortly after symptom onset, particularly in cases lacking rapid medical intervention [11,12]. A systematic review highlighted the high 30-day mortality rate post-MI, underscoring the urgency of early recognition and treatment [11]. Although significant improvements in MI incidence and treatment have been achieved, mortality remains high when symptom onset is not recognized or acted upon swiftly. As a systematic review highlights, improving symptom recognition and increasing public awareness are crucial for reducing delays in care and lowering MI mortality [13].

A further complication stems from the Increasing number of individuals surviving heart attacks but subsequently developing chronic conditions like heart failure. This phenomenon suggests that while acute mortality may be improving, the overall burden on healthcare systems is intensifying due to the rise in long-term cardiovascular complications [14,15]. This complexity underscores the importance of continuing research and public health initiatives aimed at reducing heart attack incidence and improving post-event survival.

The evaluation of MI has traditionally relied on techniques such as electrocardiography (ECG) and echocardiography (ECHO) [16]. However, these methods have notable limitations and are increasingly complemented or supplanted by novel biomarkers such as B-type natriuretic peptide (BNP), troponin, and creatine kinase isoenzyme MB (CK-MB) [17,18]. For instance, up to 10% of MI patients may present with a normal ECG, leading to false negatives [19]. Furthermore, the accuracy of ECG interpretation heavily relies on the clinician’s experience and the time available for assessment, especially in emergency settings where rapid decision making is crucial [20]. Manual echocardiogram analysis may introduce errors, with studies reporting up to 30% inaccuracy due to subjective interpretation [21]. This variability can lead to misdiagnosis, ultimately delaying necessary interventions. Despite their role in the initial assessment of heart conditions, both ECG and ECHO often have limitations in sensitivity and specificity, particularly in certain populations such as those with atypical presentations or specific anatomical variations.

Importantly, cardiac biomarkers have emerged as critical adjuncts to the diagnostic toolkit for MI. Troponin, a protein released upon myocardial injury, is known for high sensitivity and specificity for myocardial cell damage, and is, therefore, considered the gold standard for MI diagnosis [22]. Similarly, CK-MB is another biomarker that provides early evidence of myocardial stress. BNP is particularly promising, especially in the context of heart failure, as BNP and its N-terminal fragment (NT-proBNP) are secreted in response to ventricular pressure overload and correlate well with heart failure severity, indicating a clear link between cardiac stress and biomarker levels [23,24].

In instances of acute heart failure, elevated BNP levels can assist in distinguishing between cardiac and non-cardiac causes of dyspnea, further emphasizing the utility of biomarkers in complex diagnostic scenarios [23]. Their dynamic nature allows for monitoring the effectiveness of therapeutic interventions, facilitating timely adjustments in management [23,24]. Additionally, emerging research highlights the potential of using highly sensitive biosensors for rapid and effective measurements of these biomarkers, thereby delivering results faster than conventional methods and potentially leading to improved clinical outcomes [25]. In summary, MI continues to impose a major global health burden despite advancements in incidence reduction and survival. Given the limitations of traditional diagnostic tools like ECG and ECHO, the integration of cardiac biomarkers including troponin, CK-MB, and BNP has significantly enhanced diagnostic precision and patient management. This shift towards biomarker-based diagnostics marks a significant advance in cardiovascular medicine, aimed at improving early detection, treatment, and long-term outcomes for patients affected by MI. Nevertheless, despite these advances, further improvements in predictive accuracy remain possible, paving the way for artificial intelligence (AI)–based approaches that expand opportunities for early and accurate cardiovascular risk prediction.

AI has increasingly been applied in medicine for disease prediction and early diagnosis, offering new opportunities for precision healthcare, including cardiovascular diseases [26]. Several studies have demonstrated the utility of AI in MI, where machine learning (ML) models leveraging biomarkers such as troponin, CK-MB, ECG signals, and even advanced imaging modalities have achieved high diagnostic accuracy and reduced misclassification rates, and in some cases outperformed conventional risk scores [27]. Moreover, comprehensive reviews of ML methodologies—including topological data analysis, logistic regression with regularization, tree-based ensembles (Random Forest, Gradient Boosting, XGBoost, CatBoost), support vector machines (SVM), Naïve Bayes classifiers, and artificial neural networks (ANN) as well as deep learning (DL) architectures—have highlighted their complementary strengths and limitations in cardiovascular prediction tasks, underscoring the critical importance of aligning method selection with clinical context and dataset characteristics [28].

Building upon advances in AI, Explainable AI (XAI) represents the next level, becoming essential in healthcare applications where model transparency and interpretability are critical for clinical adoption [29]. Several methodologies have been developed to address the “black box” nature of complex ML models [29,30]. For example, SHAP (SHapley Additive exPlanations) uses game theory to assign feature importance values and provides both global and local explanations, though it requires significant computational resources [31]. LIME (Local Interpretable Model-agnostic Explanations) explains individual predictions by training local surrogate models, but its outputs can vary across similar cases [31]. SHAP and LIME are post hoc techniques that operate after model training and, while useful, can introduce additional computational cost or instability [32].

Explainable Boosting Machines (EBMs) are part of the broader family of explainable ML approaches, combining the predictive power of boosting algorithms with inherent interpretability [33]. EBMs emerge as inherently transparent models that offer a clear advancement over post hoc techniques, thereby enhancing both clinical applicability and trust in medical decision making [34,35,36]. They combine predictive accuracy with natural transparency and eliminate the need for complex post hoc explanations [36,37]. Built on generalized additive models with pairwise interactions, EBMs allow each feature’s contribution to be visualized and understood independently—an approach closely aligned with clinical reasoning [33]. Their computational efficiency, together with the ability to provide both global and local explanations simultaneously, makes them particularly suitable for medical applications where rapid decision making and regulatory compliance are critical [33]. In the context of MI, EBMs not only identify and rank key biochemical markers associated with cardiovascular risk but also clarify the direction and magnitude of each variable’s contribution in a human-understandable manner [38]. By striking an effective balance between predictive accuracy and interpretability, EBMs enhance clinician confidence and offer a robust framework for integrating explainable machine learning into clinical practice.

The primary objective of this study is to harness the power of EBMs to analyze a cardiovascular dataset and identify the most relevant biomarkers linked to MI risk. Unlike traditional black-box models, the use of EBM allows for the examination of complex non-linear relationships between clinical features while preserving interpretability—thus facilitating adoption in clinical environments. Ultimately, this approach seeks to provide not only a more accurate prediction framework but also novel insights into the pathophysiology of MI, supporting future diagnostic and therapeutic strategies grounded in data-driven evidence.

## 2. Materials and Methods

### 2.1. Dataset

The dataset utilized in this study comprises clinical information from individuals diagnosed with or without a history of MI. The data were originally collected in 2018 at a cardiology center located in the Erbil region of Iraq and have been previously validated in cardiovascular research [39]. This dataset was selected over alternative MI datasets for several compelling reasons that ensure both clinical relevance and methodological rigor.

The dataset contains eight carefully selected input features that represent the most critical clinical and biochemical indicators routinely used in emergency cardiac assessment: Age (years), Blood glucose level (mg/dL), Heart rate (bpm), Systolic blood pressure (mmHg), Diastolic blood pressure (mmHg), Creatine kinase-MB (CK-MB, kU/L), and Troponin level (ng/mL). These variables align with established clinical guidelines for MI diagnosis and risk stratification, ensuring that our predictive models are grounded in clinically meaningful parameters. With 1319 patient records, the dataset provides sufficient statistical power for robust model development while remaining computationally manageable, exceeding the minimum requirements for reliable machine learning model training in medical applications.

The selection of this particular dataset was further justified through comparison with alternative MI datasets available in the literature. The Cleveland Heart Disease Dataset [40], though widely used, focuses on coronary artery disease rather than acute MI and lacks specific biomarkers like troponin and CK-MB that are crucial for acute MI diagnosis. The Framingham Heart Study Dataset [41], while comprehensive, is primarily designed for long-term cardiovascular risk prediction rather than acute MI detection and may not include the specific biochemical markers essential for immediate clinical decision making. The MIMIC-III Database [42], though extensive, contains numerous variables that may introduce noise and complexity without necessarily improving MI prediction accuracy, and requires significant preprocessing efforts. In contrast, our chosen dataset represents a Middle Eastern population, which is often underrepresented in cardiovascular research dominated by Western cohorts, enhancing the generalizability of findings to broader populations.

The dataset structure comprises eight input features and one binary output variable representing the presence or absence of MI, categorized as positive (MI present) or negative (no MI). The input variables were selected based on their established clinical significance in cardiovascular assessment and their availability in typical emergency department settings, ensuring practical applicability of the resulting predictive models. Additionally, the dataset has undergone rigorous quality control measures, with complete records for all variables and standardized measurement units, reducing the need for extensive preprocessing and missing data imputation. This combination of clinical relevance, statistical adequacy, and practical applicability makes the dataset particularly suitable for developing robust and clinically implementable MI prediction models.

### 2.2. Explainable Boosting Machines (EBMs)

XAI has gained prominence for enhancing model interpretability, particularly in complex and high-stakes environments. Among various methodologies developed in this field, EBMs exemplify a unique integration of interpretability with robust predictive performance. EBMs harness the principles of Generalized Additive Models (GAMs) within a gradient boosting framework, allowing for flexible yet interpretable models that elucidate the relationship between inputs and outputs.

At the core of EBMs is their use of additive models, where the effects of predictor variables on the response are modeled independently, allowing for clear interpretability [43]. This approach yields high-performing models. It also provides insights into how individual predictors contribute, which is crucial in healthcare [44,45]. The gradient boosting aspect of EBMs helps in improving prediction accuracy by combining multiple weak learners, where each new model is trained to correct errors from the previous ones [43,46]. This approach successfully balances the trade-off between model complexity and interpretability, a significant challenge in AI deployment, particularly in sensitive applications like medical diagnosis [45].

Moreover, EBMs differ from many traditional black-box models in that they fundamentally prioritize interpretability without sacrificing accuracy. This feature is particularly pertinent when it comes to explaining complex interactions within datasets, such as ecological models predicting obesity prevalence [47,48]. In comparative studies, EBMs have demonstrated reliable performance relative to other models, such as random forests, while maintaining the ability to articulate the decision-making process in an interpretable manner [49]. By employing EBMs, stakeholders can gain insights into the models’ predictive characteristics, promoting collaboration between technical developers and domain experts to align model outcomes with real-world implications [50,51].

The significance of EBMs extends beyond mere explainability; they embody a framework for responsible AI that considers ethical implications and stakeholder engagement [44]. By providing interpretable predictions, EBMs facilitate informed decision-making processes in critical real-world applications. This transparency is essential in fostering user trust and understanding, especially in contexts where algorithmic decisions must be justified to affected individuals or societies [47,52]. In conclusion, EBMs represent a significant advancement in the quest for interpretable machine learning models. They encapsulate the need for predictive accuracy while ensuring that stakeholders can understand and trust AI outcomes, making them a pivotal component in the broader landscape of explainable AI methodologies.

### 2.3. Modeling Section

#### 2.3.1. Mathematical Framework and Modeling Approach

##### Dataset Characteristics and Preprocessing

The dataset used in the study consists of 1319 participants, including 500 healthy controls (39%) and 806 MI patients (61%). The dataset was split into 80% training and 20% test sets using random sampling with stratification to maintain class distribution. Categorical variables were converted to numerical format using one-hot encoding method, and min-max normalization was applied for continuous variables to ensure numerical stability and optimal model performance.

##### EBM Architecture

To meet the explainability requirement in clinical decision support systems, the EBM model was preferred over traditional black-box approaches. EBM was implemented using Microsoft’s InterpretML library and works based on the principle of GAMs, capable of calculating the independent contribution of each feature while maintaining high predictive accuracy.

##### Mathematical Model Formulation

The EBM model follows an additive structure mathematically expressed as:

f(x) = β_0_ + Σ_i = 1_^p^ f_i_(x_i_)

where:

- f(x) represents the final prediction score in log-odds scale for binary classification;
- β_0_ is the intercept term capturing baseline risk across the population;
- p denotes the total number of features (p = 8 clinical variables in this study);
- f_i_(x_i_) represents the additive component function for feature i, learned independently through gradient boosting;
- Each f_i_ captures non-linear relationships while maintaining interpretability.

##### Model Configuration and Hyperparameters

The model was trained with the following hyperparameters: maximum iteration count 5000, learning rate 0.01, validation rate 15%, early stopping 50 iterations, interaction term count 0 (interactions = 0), and random seed 42 for reproducibility. The training process was performed using gradient boosting methodology, and cross-validation methods were applied to prevent overfitting and ensure model generalization.

##### Interpretability-Focused Training Strategy

The model was trained without interaction terms (main effects only), aiming to clearly visualize the individual contribution of each feature and maximize the interpretability of the model. This approach enabled clear evaluation of the independent contribution of each clinical parameter to MI diagnosis, which is crucial for clinical acceptance and trust in AI-driven diagnostic systems.

##### Feature Importance Quantification

Feature importance scores were calculated using the mathematical formulation:

Importance_i = (1/n)Σ_j = 1_^n^|f_i_(x_j_)|

where:

- Importance_i quantifies the average absolute contribution of feature i across all observations;
- n represents the total number of patients in the dataset (n = 1306);
- |f_i_(x_j_)| denotes the absolute magnitude of feature i’s contribution for patient j;
- This metric provides a direct measure of each biomarker’s diagnostic significance.

##### Partial Dependence Analysis

To understand the individual effect of each feature on model predictions while controlling for other variables, partial dependence functions were computed as:

PD_j_(x_j_) = (1/n)Σ_i_ = _1_^n^f(x_j_, x_™j_^(i)^)

where:

- PD_j_(x_j_) represents the partial dependence of feature j at specific value x_j_;
- x_™__j_^(i)^ denotes all other features (excluding j) for the i-th observation;
- f(x_j_, x_−j_^(i)^) is the model prediction when feature j is fixed at x_j_ while other features vary across observations;
- This formulation isolates the effect of individual biomarkers by marginalizing out the influence of all other variables.

##### Performance Evaluation Framework

Receiver Operating Characteristic—Area Under Curve (ROC-AUC), accuracy, sensitivity/recall, specificity, precision/positive predictive value, F1 score, Matthews correlation coefficient (MCC), and balanced accuracy metrics were used to evaluate model performance. The mathematical formulations for key metrics include:

- AUC Calculation: AUC = ∫_0_^1^ TPR(FPR) d(FPR);
- Accuracy: (TP + TN)/(TP + TN + FP + FN);
- Sensitivity/Recall: TP/(TP + FN);
- Specificity: TN/(TN + FP);
- Precision/PPV: TP/(TP + FP);
- F1 score: 2 × (Precision × Recall)/(Precision + Recall).

where TP, TN, FP, and FN represent true positives, true negatives, false positives, and false negatives, respectively.

##### Clinical Interpretability Integration

The mathematical framework enables three levels of clinical interpretability:

- Global Interpretability: Feature importance ranking based on average absolute contributions across all patients;
- Feature-level Interpretability: Partial dependence plots revealing non-linear relationships between biomarkers and MI risk;
- Local Interpretability: Individual prediction explanations decomposing patient-specific biomarker contributions.

##### Mathematical Validation and Quality Assurance

Model reliability was ensured through comprehensive mathematical validation including:

- Bootstrap confidence intervals for performance metrics;
- Cross-validation assessment for generalization capability.

##### Computational Environment and Software Tools

The proposed system was implemented using Python 3.11.13 (Anaconda distribution) in Jupyter Notebook environment (version 7.4.4), which provided an interactive development platform for iterative testing and real-time result visualization. The implementation utilized several key Python libraries including pandas (v2.3.1) and numpy (v1.26.4) for data manipulation and numerical computations, scikit-learn (v1.7.0) for machine learning algorithms and model evaluation metrics, and matplotlib (v3.10.3) and seaborn (v0.13.2) for data visualization and statistical plotting. For explainable AI implementation, we employed the InterpretML library (v0.7.2) with Explainable Boosting Classifier, which enabled both high-performance predictions and interpretable model explanations. The interpret library’s InlineProvider was configured to facilitate seamless visualization of model interpretations within the Jupyter environment.

### 2.4. Ethical Considerations

Ethical review and approval were waived for this study because it used an open-access, anonymized dataset collected and publicly shared for research purposes. The dataset contains no personally identifiable information, and no human participants were directly involved by the authors. Therefore, ethical approval was not required.

## 3. Results

### 3.1. Overall Model Performance for EBM (Without Interaction Terms)

**Figure 1:**
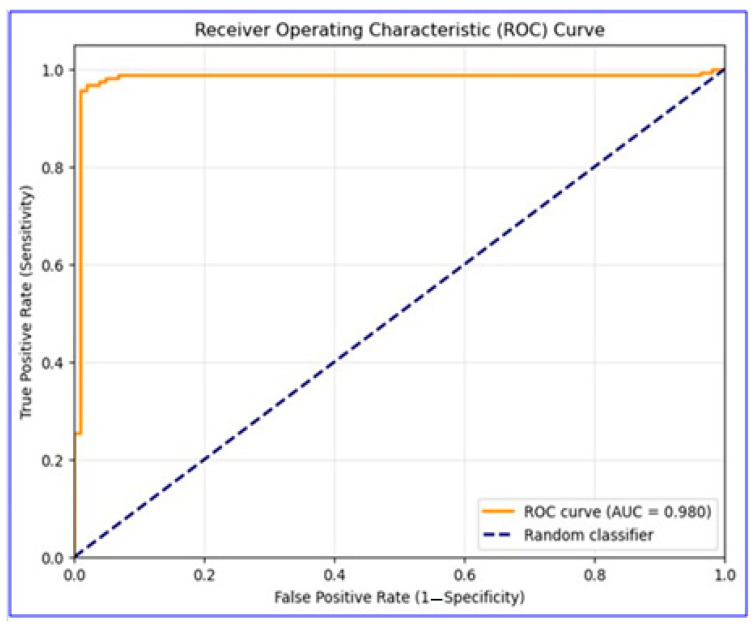
ROC curve for EBM model performance in MI diagnosis. This figure displays the overall discriminative ability of the model on the test dataset.

The EBM model developed in this study showed excellent performance in MI diagnosis. In the evaluation performed on the test dataset, the model exhibited near-perfect discriminative power with an AUC value of 0.980. ROC curve analysis (Figure 1) demonstrated excellent diagnostic performance. The curve’s trajectory near the upper left corner indicated high sensitivity and specificity achieved simultaneously.

**Figure 2:**
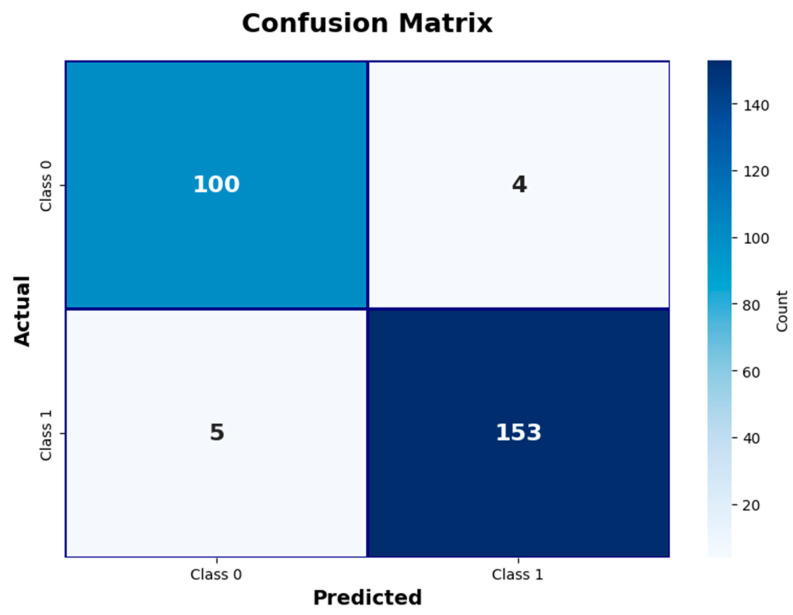
Confusion matrix illustrating EBM model classification results. This figure shows counts of true positive, true negative, false positive, and false negative cases.

The classification performance on the test dataset was analyzed in detail with the confusion matrix (Figure 2), and it was observed that the model detected both positive and negative cases with high accuracy, with false positive and false negative rates at minimal levels. These results highlight the model’s strong ability to distinguish between true positive and true negative cases.

#### 3.1.1. Detailed Performance Analysis

The detailed performance metrics presented in Table 1 suggest the model’s potential for clinical application. The model offers high reliability with a 96.6% overall accuracy rate and correctly detects the vast majority of MI cases with a 96.8% sensitivity value. The 96.2% specificity rate indicates that false positive diagnoses are minimized.

#### 3.1.2. Feature Importance Analysis

**Figure 3:**
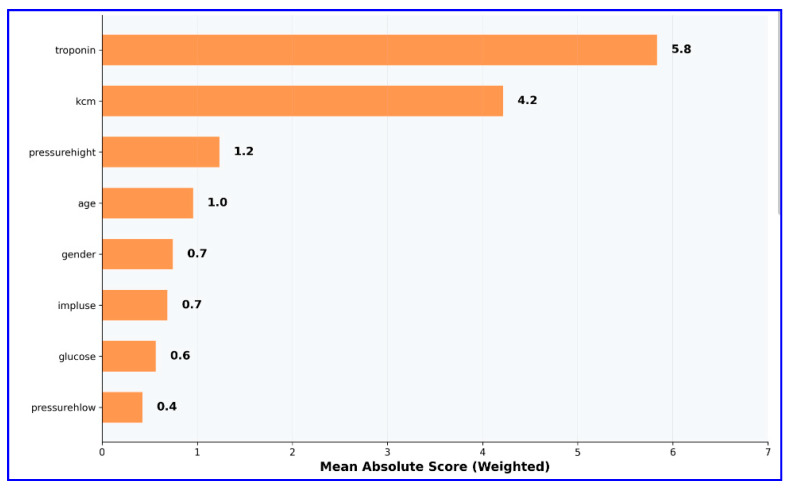
Global feature importance scores in EBM model (without interaction terms). This figure highlights the relative importance of clinical and biochemical variables.

Global feature importance scores analysis revealed the superior value of cardiac biomarkers in MI diagnosis (Figure 3).

Troponin emerged as the most influential predictor, with an importance score of 5.8, reaffirming its well-established role as the gold standard biomarker for the diagnosis of MI. CK-MB followed as the second most important variable, with an importance score of 4.2, underscoring its complementary diagnostic value in quantifying the extent of myocardial necrosis. In contrast, conventional cardiovascular risk factors—including age, gender, and vital signs such as blood pressure parameters and heart rate—exhibited minimal importance scores. This pattern clearly demonstrates that cardiac-specific biomarkers provide substantially greater diagnostic utility than demographic or hemodynamic variables, thereby reinforcing the clinical relevance and robustness of biomarker-driven models in MI detection.

#### 3.1.3. Partial Dependence Analyses

Leveraging the explainability advantage of the EBM model, partial dependence analyses were performed for the three variables with the highest importance scores (troponin, CK-MB, and systolic blood pressure). These analyses visualized the independent effect of each variable on model predictions, revealing the non-linear character of clinical parameters’ contributions to MI diagnosis. The partial dependence plots revealed the marginal effect of each feature on the model output while keeping other variables constant, providing important insights into how these parameters should be interpreted in the clinical decision-making process.

**Figure 4:**
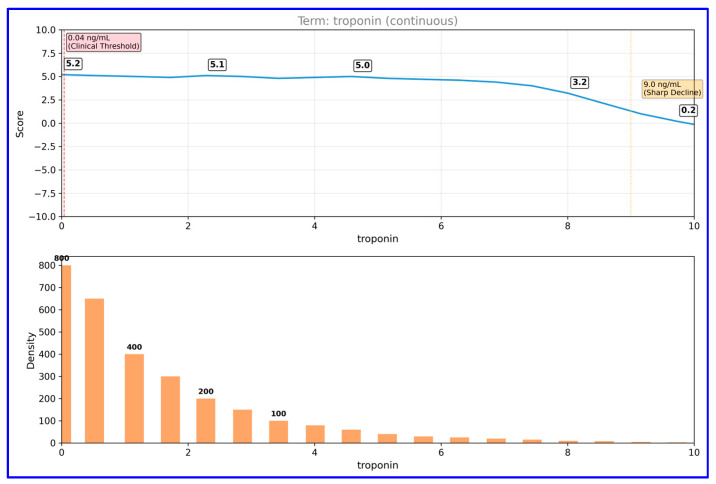
Partial dependence plot for troponin in EBM model. This figure depicts the non-linear relationship between troponin levels and predicted MI risk.

Partial dependence analysis for troponin (Figure 4) revealed the non-linear character of this biomarker’s effect on the model.

**Figure 5:**
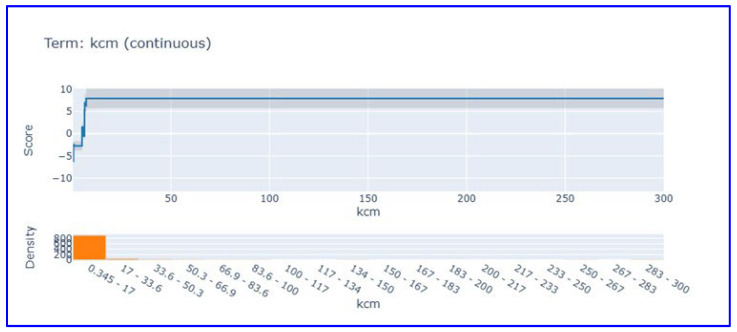
Partial dependence plot for CK-MB in EBM model. This figure shows threshold behavior and contribution of CK-MB levels to MI prediction.

Troponin levels remained stable at a positive score (~5) between 0–9 ng/mL, but declined sharply above this threshold. Data distribution analysis shows that the majority of samples are concentrated at low troponin values (0–2 ng/mL), confirming that troponin levels in the normal population are generally low. CK-MB partial dependence analysis (Figure 5) showed that this parameter exhibited a threshold effect.

CK-MB, which shows negative scores at low values (0–20 U/L), shows a sharp increase after approximately 20 U/L threshold value, reaching a positive plateau (~7) and remaining stable at this level. This pattern scientifically supports the use of cut-off values for CK-MB in clinical diagnosis.

**Figure 6:**
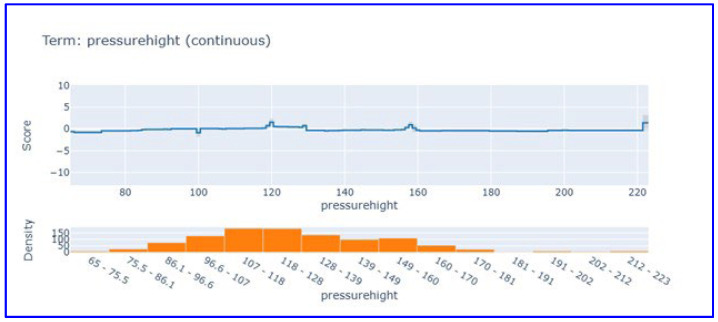
Partial dependence plot for systolic blood pressure in EBM model. This figure visualizes the limited influence of systolic blood pressure on model predictions.

Systolic blood pressure partial dependence analysis (Figure 6) showed that this parameter makes limited contribution to the model score. Systolic blood pressure, which exhibits a score close to zero and stable in the 65–220 mmHg range, revealed that it is not a discriminative feature in heart attack diagnosis. This finding supports that vital signs have limited diagnostic value compared to cardiac biomarkers in acute MI diagnosis.

#### 3.1.4. Local Explainability Analyses

##### Negative Case Analysis (Patient 1)

**Figure 7:**
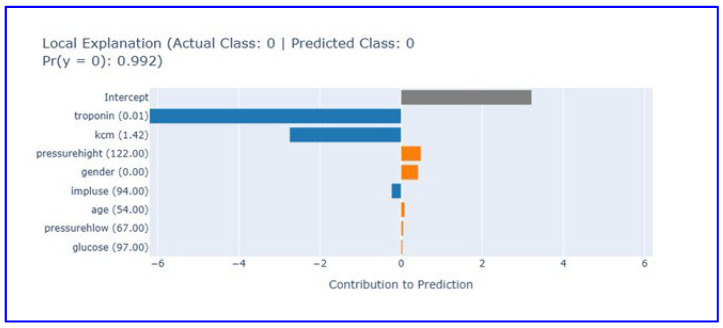
Local explanation plot for a true negative case (Patient 1). This figure explains how low biomarker values led the model to predict absence of MI. (Orange bars: Features contributing positively to the prediction, blue bars: Features contributing negatively to the prediction, gray bar: Intercept (baseline)).

Local explanation analysis for the first patient (Figure 7) demonstrated the model’s ability to make correct negative diagnoses.

In this case where the actual and predicted class was 0 (no heart attack), the model reached the correct diagnosis with 99.2% confidence level. Low troponin (0.01) and CK-MB (1.42) values provided strong negative contribution, supporting the absence of myocardial damage. Other parameters showed limited contribution, emphasizing the diagnostic superiority of cardiac biomarkers. While systolic blood pressure (122 mmHg) and gender (0.0) showed minimal positive contribution, pulse (94/min), age (54 years), diastolic blood pressure (67 mmHg), and glucose (97 mg/dL) values made near-zero contributions. The intercept value contributes roughly 3.5 units to the baseline prediction. This analysis shows how effective low cardiac biomarker levels are in excluding heart attack diagnosis and how the EBM model reached the correct diagnosis for this patient.

##### Positive Case Analysis (Patient 4)

**Figure 8:**
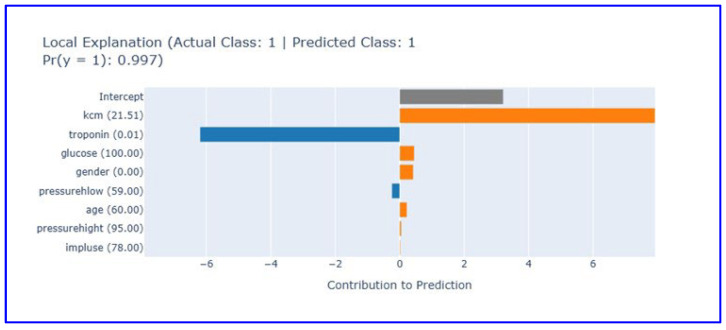
Local explanation plot for a true positive case (Patient 4). This figure illustrates how elevated CK-MB levels drove the model’s MI prediction despite low troponin. (Orange bars: Features contributing positively to the prediction, blue bars: Features contributing negatively to the prediction, gray bar: Intercept (baseline)).

Local explanation analysis for the fourth patient (Figure 8) revealed the model’s capacity for correct positive diagnosis.

**Figure 9:**
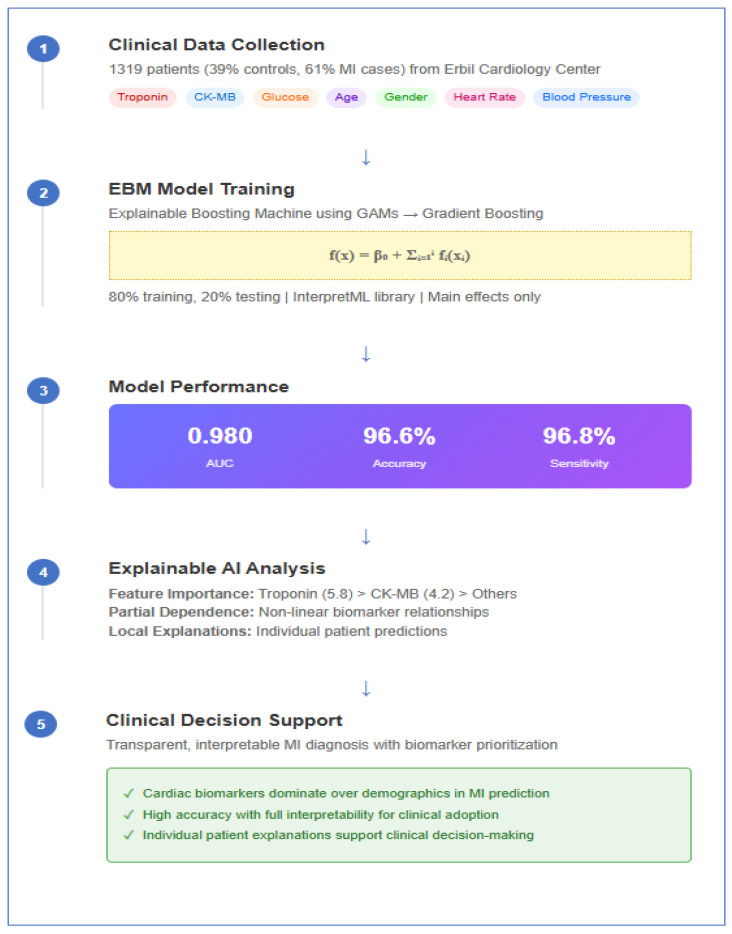
The EBM system architecture for MI diagnosis.

The local explanation plot illustrates the EBM model’s decision-making process for a correctly classified positive case (actual class: 1, predicted class: 1) with 99.7% confidence. Elevated CK-MB levels (21.51) provide the strongest positive contribution (5) to the prediction, serving as the primary indicator of myocardial necrosis. Despite low troponin levels (0.01) contributing negatively to the prediction (−4), the model successfully integrates multiple biomarker information to reach the correct diagnosis. Other clinical parameters including glucose (100 mg/dL), gender (0.0), diastolic blood pressure (59 mmHg), age (60 years), systolic blood pressure (95 mmHg), and heart rate (78/min) show limited contributions. The intercept provides a baseline contribution of approximately 3.5 units. The EBM system architecture for MI diagnosis is shown in Figure 9.

### 3.2. Overall Model Performance for EBM (With Interaction Terms)

To address concerns regarding the exclusion of interaction terms, preprocessing steps and feature sets were kept the same as in the interaction-free model, and an additional analysis was performed using interaction-enabled EBM to observe the interaction effects of variables. Including interaction terms enabled the algorithm to account for binary feature relationships (e.g., biomarker-hemodynamic interactions) that could contribute to MI prediction.

**Figure 10:**
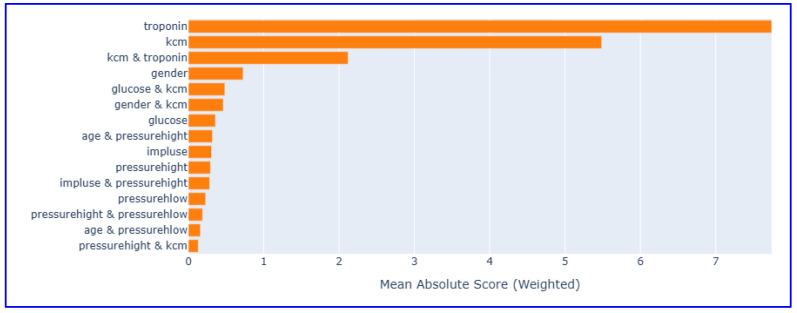
Global feature importance scores in EBM model (with interaction terms).

When constructing the interaction-based EBM, a systematic grid search analysis was performed using 5-fold cross-validation testing different interaction configurations (0, 1, 2, 3, 5, 8, 10 interactions). Cross-validation theoretically determined 8 interactions as optimal. The performance metrics obtained from the interactive model and the non-interactive model are presented in Table 2, while the global variable importance graph obtained from the interactive model is presented in Figure 10.

The interaction-based EBM maintained excellent overall performance, achieving high accuracy, AUC, and balanced accuracy scores with stable sensitivity and specificity. These results demonstrate that adding interaction effects does not lead to a significant improvement in performance metrics (only changes in error distribution) and support the idea that simple, interpretable models can be as effective as complex interactive approaches in cardiovascular risk assessment.

### 3.3. Comparative Analysis Against Existing Machine Learning Approaches

The dataset was split into training (80%) and test (20%) subsets using a stratified split with a fixed random seed (42) to ensure reproducibility. We compared a transparent EBM with five widely used baselines: Logistic Regression (LR), Support Vector Machine (SVM), Neural Network (MLP), Naive Bayes (GaussianNB), and k-Nearest Neighbors (k-NN). Model configurations were as follows:

R: solver = “liblinear”, C = 0.3, max_iter = 200, random_state = 42.

SVM (RBF): kernel = “rbf”, C = 0.5, gamma = “scale”, probability = True, random_state = 42.

MLP: hidden_layer_sizes = (15, 8), alpha = 0.01, learning_rate_init = 0.01, max_iter = 150, random_state = 42.

GaussianNB: var_smoothing = 1 × 10^−8^.

k-NN: n_neighbors = 7, weights = “uniform”.

All models were trained on the same training set and evaluated on the held-out test set. The models were evaluated using the ROC curve (AUC), accuracy, precision (PPV), recall (sensitivity), specificity, F1 score, negative predictive value (NPV), balanced accuracy, and Matthews correlation coefficient (MCC).

**Figure 11:**
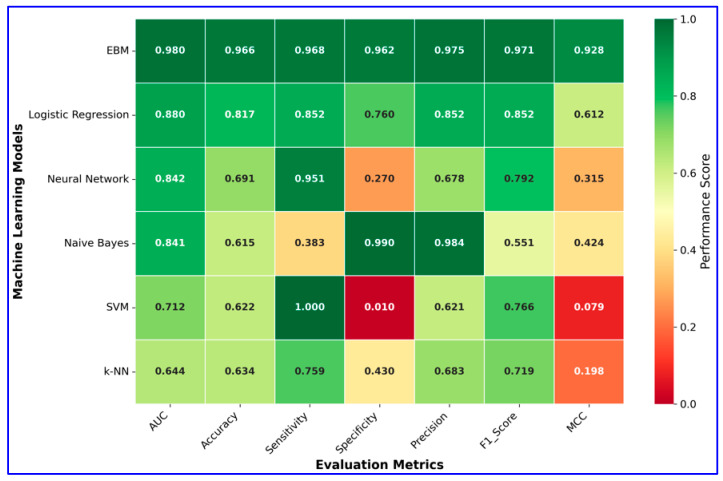
Heatmap visualization of model performance metrics.

The values for the performance metrics of the models are given in Table 3. The values for the performance criteria of the models are given in Table 3 and their graphical representation is presented in Figure 11.

According to the comparative results presented in Table 3, the EBM substantially outperformed all baseline models across nearly all evaluation metrics. EBM achieved the highest accuracy (96.6%), balanced accuracy (96.5%), and AUC (0.98), with excellent precision (97.5%), sensitivity (96.8%), and specificity (96.2%). In contrast, traditional classifiers such as Logistic Regression, Neural Network, Naive Bayes, SVM, and k-NN showed markedly lower overall performance, particularly in balanced accuracy and MCC. These findings highlight the superiority of EBM in providing both robust predictive power and reliability in MI diagnosis.

## 4. Discussion

MI remains a leading cause of mortality globally, characterized by severe and potentially lethal arrhythmias due to acute ischemic episodes that disrupt the normal electrical function of the heart [53]. The lethality associated with MI can be significantly exacerbated by multiple clinical factors, including advanced age, the extent of myocardial injury, and the emergence of complications such as ventricular tachycardia or heart failure. These conditions collectively elevate the risk of sudden cardiac death, particularly in the acute and subacute phases following MI [54,55]. Specific cardiac conditions, such as hypertensive heart disease and ischemia-induced lethal arrhythmias, also contribute significantly to this risk, showcasing the multifaceted nature of myocardial infarctions and their outcomes [56].

In terms of diagnostics, ECG remains a cornerstone for the assessment of MI, particularly in detecting acute ischemic changes. However, the diagnostic efficacy of ECG can be limited due to patient-specific variability in electrical manifestations—especially in cases of subtle or atypical MI presentations. As a result, sole reliance on ECG may lead to misinterpretations or missed diagnoses, particularly in emergency settings where time constraints and clinician experience vary significantly [57]. This limitation has prompted a shift towards biomarkers such as BNP, troponin, and CK-MB, which provide more reliable indicators of myocardial injury and heart failure, particularly in patients where ECG results are inconclusive. Biomarkers have shown promise in not only diagnosing MI but also in predicting adverse cardiovascular events, thus influencing therapeutic decisions and improving patient outcomes [58,59,60].

In summary, the complexities of MI’s lethality and the evolution of diagnostic methods highlight the need for integrating traditional heart rhythm monitoring and innovative biomarker approaches in clinical practice. This hybrid model aims to provide comprehensive patient management during acute coronary events while addressing the significant challenges posed by both the infarction and its complications.

Recognizing the limitations of conventional diagnostic tools and the multifactorial etiology of MI, the present study integrates EBMs as a novel interpretable machine learning method into the analysis of routinely collected clinical data. EBM, situated within the paradigm of XAI, provides a critical advantage by combining high predictive accuracy with full model transparency [61]. This ensures that each variable’s contribution to prediction outcomes can be explicitly interpreted, a feature that is essential for clinical implementation and trust. Unlike black-box models, EBM maintains the interpretability necessary for physicians to validate and contextualize machine-generated risk assessments within clinical decision-making processes [33].

From a scientific standpoint, this study contributes to the literature by demonstrating how explainable machine learning approaches can be effectively used to uncover key cardiac biomarkers such as troponin and CK-MB and assess their relative importance in predicting MI. By employing EBM on a well-characterized cardiovascular dataset, the study offers not only a practical framework for clinical prediction, it also provides insights into the pathophysiological mechanisms underpinning acute coronary syndromes. Moreover, this work bridges a significant gap in existing research by operationalizing an interpretable AI model that respects the diagnostic complexity of MI, thereby promoting transparency, reproducibility, and applicability in real-world healthcare settings.

In light of the findings obtained from this study, the EBM model demonstrated excellent performance in the diagnosis of MI, suggesting its high potential for clinical implementation. With an AUC of 0.980 and an overall accuracy of 96.6%, the model provided high discrimination between patients with and without MI. This level of performance, combined with interpretability, positions EBM as a valuable candidate for decision support systems in cardiology.

The model’s sensitivity (96.8%) and specificity (96.2%) indicate that it is highly capable of detecting true positives while minimizing false alarms—a balance that is crucial in acute cardiac settings. Moreover, the Matthews Correlation Coefficient (0.928) and F1 score (0.971) further confirm the robustness and reliability of the classification. Importantly, the intercept in the EBM model contributed approximately 3.5 units to the baseline prediction, reflecting an inherent risk level across the cohort. This finding aligns the statistical outputs with clinical intuition, suggesting that even in the absence of strong predictive features, there remains a baseline risk that must be accounted for in MI prediction models.

A particularly notable outcome of the study is the significant diagnostic value of cardiac-specific biomarkers, especially troponin and CK-MB, compared to demographic and hemodynamic features such as age, blood pressure, and heart rate. Troponin emerged as the most important feature, consistent with its role as the gold standard in MI diagnosis. CK-MB also displayed strong predictive value, supporting its clinical relevance. In contrast, traditional risk indicators like systolic blood pressure contributed limitedly, aligning with emerging perspectives that emphasize biochemical markers over vital signs in acute MI detection [58,62,63,64]. An additional consideration pertains to the negative contributions observed for low biomarker levels, particularly troponin and CK-MB. While such negative contributions enhance the model’s ability to correctly identify true negatives, they also highlight a potential vulnerability with respect to false negatives. Specifically, patients presenting early after symptom onset or those with atypical MI may initially exhibit low or near-normal biomarker levels. In these scenarios, the model’s reliance on strong negative contributions could result in underestimation of risk and misclassification. This phenomenon mirrors well-documented clinical challenges in which biochemical markers fail to rise promptly during the early window of myocardial injury, necessitating repeat testing and multimodal assessment. Therefore, although the EBM model demonstrated robust performance, the negative contributions of low biomarker values should be interpreted cautiously in clinical practice, and future studies should integrate serial biomarker measurements or complementary modalities (e.g., ECG or imaging) to mitigate the risk of false negatives.

The partial dependence analyses provided additional insights into the non-linear and threshold-based effects of key biomarkers. For example, troponin’s influence remained stable at low levels but declined after a certain point, while CK-MB displayed a sharp increase in contribution beyond the 20 U/L threshold. These findings not only validate known clinical cut-offs but also underscore the interpretive strength of EBM in capturing complex, non-linear relationships [65,66].

Furthermore, local explanation plots illustrated the model’s decision-making logic at the individual level, confirming that it could correctly diagnose both negative and positive cases with high confidence. In the case of a true negative, low levels of troponin and CK-MB were instrumental in ruling out MI, while in a true positive case, elevated CK-MB levels compensated for normal troponin readings, highlighting the model’s capability to integrate multiple signals effectively.

Overall, the results of this study underscore the promise of explainable machine learning approaches like EBM in enhancing diagnostic accuracy while maintaining transparency—a key requirement in clinical environments. Future work should explore the integration of such models into real-time healthcare systems and assess their utility in diverse patient populations to further validate their generalizability and clinical impact.

In doing so, the findings presented here extend beyond conventional model performance metrics; they underscore the potential of EBM not only as a high-performing classifier but also as a powerful instrument for biomarker prioritization and clinical risk stratification in the context of MI. By providing interpretable insights into the individual and collective contributions of diagnostic variables, the EBM model facilitates a more nuanced understanding of disease signatures, which is central to both evidence-based practice and precision cardiology.

Moreover, this study contributes to the broader discourse on responsible and transparent AI in healthcare by demonstrating how interpretable models can support ethically sound, clinically meaningful decision making. The integration of explainable AI tools such as EBM into diagnostic pathways resonates with the core principles of precision medicine—tailoring interventions to individual patient profiles—and reflects a paradigm shift toward more accountable machine learning applications. As such, the methodology and results of this work align with contemporary efforts to bridge data science and clinical practice, paving the way for future research that balances predictive power with interpretability and clinical utility.

### Limitations

Despite the promising results, several limitations of this study must be acknowledged. **(i)** First, the analysis was based on a single-center, open-access dataset collected from a specific regional population. While this dataset provides transparency, reproducibility, and accessibility, it inherently reflects local healthcare practices and demographic characteristics, which may not fully represent the diversity encountered in broader clinical environments. Consequently, the model’s generalizability to different healthcare systems, ethnic groups, and patient subpopulations remains uncertain. In real-world applications, heterogeneity in comorbidities, risk factors, and care delivery pathways can significantly influence both biomarker dynamics and model performance. This limitation underscores the need for validation across multi-center, multi-ethnic, and prospective cohorts to establish robustness and applicability in diverse clinical settings. **(ii)** Second, although the dataset includes clinically relevant features, its retrospective design restricts causal inference, and the sample size—while sufficient for internal training and testing—may not capture the full spectrum of MI presentations, particularly among underrepresented groups such as women, the elderly, or patients with atypical or silent MI. **(iii)** Third, while the EBM framework provides high interpretability, its visual explanations (partial dependence plots and local explanation outputs) may require additional training and clinical decision-support infrastructure to be integrated effectively into routine medical practice, which could pose an adoption barrier for physicians unfamiliar with AI-based models. **(iv)** Fourth, the model was trained on a limited set of eight routine biochemical and clinical parameters, excluding potentially important data sources such as imaging, electrocardiography, genetic or proteomic markers, and longitudinal biomarker measurements. The exclusion of such modalities may constrain the model’s predictive scope and limit its ability to reflect the multidimensional nature of MI diagnosis in real-world practice. **(v)** Finally, the lack of external validation represents a significant limitation. While the model demonstrated excellent internal performance (AUC 0.980, accuracy 96.6%), the absence of testing on independent, heterogeneous datasets prevents definitive conclusions about its clinical utility across different populations. Future research should aim to validate this approach in larger, multicenter, and prospective cohorts, ideally incorporating diverse demographic and clinical variables. Additionally, extending the model to integrate multimodal data (e.g., imaging, serial biomarker dynamics, detailed clinical history) may further enhance its robustness and clinical applicability.

## 5. Conclusions

This study demonstrates the effectiveness of EBM for MI diagnosis, achieving exceptional performance with an AUC of 0.980, accuracy of 96.6%, sensitivity of 96.8%, and specificity of 96.2%. The results provide strong evidence for the clinical utility of interpretable AI in cardiovascular medicine. The feature importance analysis revealed that cardiac biomarkers, specifically troponin (importance score: 5.8) and CK-MB (importance score: 4.2), significantly outperformed traditional risk factors such as age, gender, and vital signs in diagnostic value. This finding supports the paradigm shift toward biomarker-driven precision medicine in acute MI detection. Partial dependence analyses further validated clinical knowledge by demonstrating troponin’s stable contribution (~5) in the 0–9 ng/mL range and CK-MB’s threshold effect beyond 20 U/L, confirming established diagnostic cut-offs. Local explanation analyses demonstrated the model’s ability to provide confident, interpretable predictions (99.2% and 99.7% confidence levels) for individual patients, successfully integrating multiple biomarker signals to reach accurate diagnoses. The consistent intercept contribution of approximately 3.5 units reflects appropriate baseline risk assessment across the population. While the data reflect clinically relevant characteristics and are sufficient for model development, regional differences in access to healthcare, population demographics, and cardiovascular risk profiles may influence the model’s applicability in broader or more diverse settings. Therefore, findings should be interpreted with caution until the model is tested in various populations and healthcare systems. Future research should aim to replicate these results using multicenter prospective datasets and integrate additional clinical characteristics and real-time monitoring parameters to improve both performance and applicability in clinical decision support systems. External validation across diverse healthcare settings remains essential to confirm the model’s generalizability and clinical impact. The demonstrated combination of high predictive accuracy with complete interpretability positions EBM as a valuable tool for responsible AI deployment in healthcare, supporting both evidence-based practice and regulatory compliance requirements for explainable medical AI systems.

## Figures and Tables

**Table 1:** Model performance metrics and 95% confidence intervals.

Performance Metrics	Value	95% CI
Accuracy	0.966	0.944–0.988
Balanced Accuracy	0.965	0.943–0.987
MCC	0.928	0.897–0.960
PPV	0.975	0.936–0.993
Sensitivity	0.968	0.928–0.990
Specificity	0.962	0.904–0.989
F1 Score	0.971	0.951–0.992
NPV	0.952	0.892–0.984

MCC: Matthews Correlation Coefficient; PPV: Positive Predictive Value (Precision); Sensitivity: Recall; NPV: Negative Predictive Value; CI: Confidence Interval.

**Table 2:** EBM model performance comparison: baseline vs. interactions.

Metrics	Without Interaction (Baseline)	With Interaction
Accuracy	96.6%	96.6%
Precision	97.5%	96.8%
Recall/Sensitivity	96.8%	97.5%
Specificity	96.2%	95.2%
F1 Score	0.971	0.971
NPV	95.2%	96.2%

**Table 3:** The values for the performance metrics of the models.

Models	Accuracy	Balanced Accuracy	MCC	PPV	Recall	Sensitivity	Specificity	NPV	AUC
EBM	0.966	0.965	0.928	0.975	0.968	0.962	0.971	0.952	0.98
LR	0.817	0.806	0.612	0.852	0.852	0.760	0.852	0.760	0.88
NN	0.691	0.610	0.315	0.678	0.951	0.270	0.792	0.771	0.84
Naïve Bayes	0.615	0.686	0.424	0.984	0.383	0.990	0.551	0.497	0.84
SVM	0.622	0.505	0.079	0.621	1.000	0.010	0.766	1.000	0.71
k-NN	0.634	0.595	0.198	0.683	0.759	0.430	0.719	0.524	0.64

LR: Logistic Regression; NN: Neural network; SVM: Support Vector Machine; k-NN: K Nearest Neighbors; Sensitivity: Recall; PPV: Precision; MCC: Matthews Correlation Coefficient; PPV: Positive Predictive Value; NPV: Negative Predictive Value; AUC: Area Under the Curve.

## Data Availability

This study used an open-access dataset that is publicly available, and interested researchers can access it via https://doi.org/10.17632/65gxgy2nmg.2.

## References

[1] Zaheen M., Pender P., Dang Q.M., Sinha E., Chong J.J.H., Chow C.K., Zaman S. (2025). Myocardial Infarction in the Young: Aetiology, Emerging Risk Factors, and the Role of Novel Biomarkers. J. Cardiovasc. Dev. Dis..

[2] Radisauskas R., Sileikiene L., Kranciukaite-Butylkiniene D., Augustis S., Jasukaitiene E., Luksiene D., Tamosiunas A., Marcinkeviciene K., Virviciute D., Zaliaduonyte D. (2025). Trends in Myocardial Infarction Morbidity and Mortality from Ischemic Heart Disease in Middle-Aged Lithuanian Population from 2000 to 2023: Data from Population-Based Kaunas Ischemic Heart Disease Register. Medicina.

[3] Luo Y., Liu J., Zeng J., Pan H. (2024). Global burden of cardiovascular diseases attributed to low physical activity: An analysis of 204 countries and territories between 1990 and 2019. Am. J. Prev. Cardiol..

[4] Lewis E.F., Moye L.A., Rouleau J.L., Sacks F.M., Arnold J.M., Warnica J.W., Flaker G.C., Braunwald E., Pfeffer M.A. (2003). Predictors of late development of heart failure in stable survivors of myocardial infarction: The CARE study. J. Am. Coll. Cardiol..

[5] Gouda P., Savu A., Bainey K.R., Kaul P., Welsh R.C. (2021). Long-term risk of death and recurrent cardiovascular events following acute coronary syndromes. PLoS ONE.

[6] Butler J., Hammonds K., Talha K.M., Alhamdow A., Bennett M.M., Bomar J.V.A., Ettlinger J.A., Traba M.M., Priest E.L., Schmedt N. (2025). Incident heart failure and recurrent coronary events following acute myocardial infarction. Eur. Heart J..

[7] Timmis A., Townsend N., Gale C.P., Torbica A., Lettino M., Petersen S.E., Mossialos E.A., Maggioni A.P., Kazakiewicz D., May H.T. (2020). European Society of Cardiology: Cardiovascular Disease Statistics 2019. Eur. Heart J..

[8] Nichols M., Townsend N., Scarborough P., Rayner M. (2013). Cardiovascular disease in Europe: Epidemiological update. Eur. Heart J..

[9] Kivimäki M., Batty G.D., Pentti J., Shipley M.J., Sipilä P.N., Nyberg S.T., Suominen S.B., Oksanen T., Stenholm S., Virtanen M. (2020). Association between socioeconomic status and the development of mental and physical health conditions in adulthood: A multi-cohort study. Lancet. Public Health.

[10] Kristono G.A. (2021). The Effects of Health Determinants and Inequities on Acute Myocardial Infarction in New Zealand: An Epidemiological Essay. N. Z. Med. Stud. J..

[11] Johansson S., Rosengren A., Young K., Jennings E. (2017). Mortality and morbidity trends after the first year in survivors of acute myocardial infarction: A systematic review. BMC Cardiovasc. Disord..

[12] De Luca G., Suryapranata H., Ottervanger J.P., Antman E.M. (2004). Time delay to treatment and mortality in primary angioplasty for acute myocardial infarction: Every minute of delay counts. Circulation.

[13] Birnbach B., Höpner J., Mikolajczyk R. (2020). Cardiac symptom attribution and knowledge of the symptoms of acute myocardial infarction: A systematic review. BMC Cardiovasc. Disord..

[14] Conrad N., Judge A., Tran J., Mohseni H., Hedgecott D., Crespillo A.P., Allison M., Hemingway H., Cleland J.G., McMurray J.J.V. (2018). Temporal trends and patterns in heart failure incidence: A population-based study of 4 million individuals. Lancet.

[15] Gerber Y., Weston S.A., Enriquez-Sarano M., Berardi C., Chamberlain A.M., Manemann S.M., Jiang R., Dunlay S.M., Roger V.L. (2016). Mortality Associated with Heart Failure After Myocardial Infarction: A Contemporary Community Perspective. Circ. Heart Fail..

[16] Kim S.Y., Lee J.P., Shin W.R., Oh I.H., Ahn J.Y., Kim Y.H. (2022). Cardiac biomarkers and detection methods for myocardial infarction. Mol. Cell Toxicol..

[17] Katsioupa M., Kourampi I., Oikonomou E., Tsigkou V., Theofilis P., Charalambous G., Marinos G., Gialamas I., Zisimos K., Anastasiou A. (2023). Novel Biomarkers and Their Role in the Diagnosis and Prognosis of Acute Coronary Syndrome. Life.

[18] Fan J., Ma J., Xia N., Sun L., Li B., Liu H. (2017). Clinical Value of Combined Detection of CK-MB, MYO, cTnI and Plasma NT-proBNP in Diagnosis of Acute Myocardial Infarction. Clin. Lab..

[19] Taylor C., Hobbs R. (2010). Diagnosing Heart Failure—Experience and ‘Best Pathways’. Eur. Cardiol. Rev..

[20] Perrichot A., Vaittinada Ayar P., Taboulet P., Choquet C., Gay M., Casalino E., Steg P.G., Curac S., Vaittinada Ayar P. (2023). Assessment of real-time electrocardiogram effects on interpretation quality by emergency physicians. BMC Med. Educ..

[21] Wang S., Hu P. (2022). Deep Learning for Automated Echocardiogram Analysis. J. Stud. Res..

[22] Aydın S., Uğur K., Aydın S., Şahin İ., Yardım M. (2019). Biomarkers in acute myocardial infarction: Current perspectives. Vasc. Health Risk Manag..

[23] Troughton R.W., Felker G.M., Januzzi J.L. (2013). Natriuretic Peptide-Guided Heart Failure Management. Eur. Heart J..

[24] Maries L., Maniţiu I. (2013). Diagnostic and Prognostic Values of B-Type Natriuretic Peptides (BNP) and N-Terminal Fragment Brain Natriuretic Peptides (NT-pro-BNP): Review Article. Cardiovasc. J. Afr..

[25] Liu Q., Aroonyadet N., Song Y., Wang X., Cao X., Liu Y., Cong S., Wu F., Thompson M.E., Zhou C. (2016). Highly Sensitive and Quick Detection of Acute Myocardial Infarction Biomarkers Using In_2_O_3_ Nanoribbon Biosensors Fabricated Using Shadow Masks. ACS Nano.

[26] Sun X., Yin Y., Yang Q., Huo T. (2023). Artificial intelligence in cardiovascular diseases: Diagnostic and therapeutic perspectives. Eur. J. Med. Res..

[27] Patel S.J., Yousuf S., Padala J.V., Reddy S., Saraf P., Nooh A., Fernandez Gutierrez L.M.A., Abdirahman A.H., Tanveer R., Rai M. (2024). Advancements in Artificial Intelligence for Precision Diagnosis and Treatment of Myocardial Infarction: A Comprehensive Review of Clinical Trials and Randomized Controlled Trials. Cureus.

[28] Szymańska C., Baszko A. (2025). Artificial Intelligence Tools in Myocardial Infarction Prognosis: Evaluating the Performance of Machine Learning and Deep Learning Models. Curr. Cardiol. Rev..

[29] Band S., Yarahmadi A., Hsu C., Biyari M., Sookhak M., Ameri R., Dehzangi I., Chronopoulos A., Liang H. (2023). Application of explainable artificial intelligence in medical health: A systematic review of interpretability methods. Inf. Med. Unlocked.

[30] Hassija V., Chamola V., Mahapatra A., Singal A., Goel D., Huang K., Scardapane S., Spinelli I., Mahmud M., Hussain A. (2024). Interpreting Black-Box Models: A Review on Explainable Artificial Intelligence. Cogn. Comput..

[31] Hermosilla P., Berríos S., Allende-Cid H. (2025). Explainable AI for Forensic Analysis: A Comparative Study of SHAP and LIME in Intrusion Detection Models. Appl. Sci..

[32] Velmurugan M., Ouyang C., Sindhgatta R., Moreira C. (2025). Through the looking glass: Evaluating post hoc explanations using transparent models. Int. J. Data Sci. Anal..

[33] Lolak S., Attia J., McKay G.J., Thakkinstian A. (2023). Comparing Explainable Machine Learning Approaches with Traditional Statistical Methods for Evaluating Stroke Risk Models: Retrospective Cohort Study. JMIR Cardio.

[34] Sharma N.A., Chand R.R., Buksh Z., Ali A.B.M.S., Hanif A., Beheshti A. (2024). Explainable AI Frameworks: Navigating the Present Challenges and Unveiling Innovative Applications. Algorithms.

[35] Khattak A., Zhang J., Chan P.-W., Chen F., Almujibah H. (2024). Explainable Boosting Machine: A Contemporary Glass-Box Strategy for the Assessment of Wind Shear Severity in the Runway Vicinity Based on the Doppler Light Detection and Ranging Data. Atmosphere.

[36] Chen Z., Tan S., Nori H., Inkpen K., Lou Y., Caruana R. Using Explainable Boosting Machines (EBMs) to Detect Common Flaws in Data. Proceedings of the Machine Learning and Principles and Practice of Knowledge Discovery in Databases.

[37] Mahamadou A.J.D., Rodrigues E.A., Vakorin V., Antoine V., Moreno S. (2025). Interpretable machine learning for precision cognitive aging. Front. Comput. Neurosci..

[38] Arslan A.K., Yagin F.H., Algarni A., Al-Hashem F., Ardigò L.P. (2024). Combining the Strengths of the Explainable Boosting Machine and Metabolomics Approaches for Biomarker Discovery in Acute Myocardial Infarction. Diagnostics.

[39] Maghdid S., Rashid T. (2022). An Extensive Dataset for the Heart Disease Classification System. https://data.mendeley.com/datasets/65gxgy2nmg/2.

[40] Janosi A., Steinbrunn W., Pfisterer M., Detrano R. https://www.kaggle.com/datasets/ritwikb3/heart-disease-cleveland.

[41] Bhardwaj A. https://www.kaggle.com/datasets/aasheesh200/framingham-heart-study-dataset.

[42] Johnson A., Pollard T., Mark R. https://physionet.org/content/mimiciii/1.4/.

[43] Maillart A., Robert C.Y. (2024). Distill Knowledge of Additive Tree Models into Generalized Linear Models: A New Learning Approach for Non-Smooth Generalized Additive Models. Ann. Actuar. Sci..

[44] Linardatos P., Papastefanopoulos V., Kotsiantis S. (2020). Explainable AI: A Review of Machine Learning Interpretability Methods. Entropy.

[45] Burkart N., Huber M.F. (2021). A Survey on the Explainability of Supervised Machine Learning. J. Artif. Intell. Res..

[46] Arrieta A.B., Díaz-Rodríguez N., Ser J.D., Bennetot A., Tabik S., Barbado A., García S., Gil-López S., Molina D., Benjamins R. (2020). Explainable Artificial Intelligence (XAI): Concepts, Taxonomies, Opportunities and Challenges Toward Responsible AI. Inf. Fusion.

[47] Allen B. (2023). An Interpretable Machine Learning Model of Cross-Sectional U.S. County-Level Obesity Prevalence Using Explainable Artificial Intelligence. PLoS ONE.

[48] Allen B., Lane M., Steeves E.A., Raynor H.A. (2022). Using Explainable Artificial Intelligence to Discover Interactions in an Ecological Model for Obesity. Int. J. Environ. Res. Public Health.

[49] Hernández M., Ramon-Julvez U., Viladés E., Ciordía B.C., Mayordomo E., García-Martín E. (2023). Explainable Artificial Intelligence Toward Usable and Trustworthy Computer-Aided Diagnosis of Multiple Sclerosis from Optical Coherence Tomography. PLoS ONE.

[50] Marcinkevičs R., Vogt J.E. (2020). Interpretability and Explainability: A Machine Learning Zoo Mini-Tour. arXiv.

[51] Murdoch W.J., Singh C., Kumbier K., Abbasi-Asl R., Yu B. (2019). Definitions, Methods, and Applications in Interpretable Machine Learning. Proc. Natl. Acad. Sci. USA.

[52] Scheinker D., Valencia A., Rodríguez F. (2019). Identification of Factors Associated with Variation in US County-Level Obesity Prevalence Rates Using Epidemiologic vs Machine Learning Models. JAMA Netw. Open.

[53] Chen H. (2013). Sudden Cardiac Death in a Case of Non-Dominant Coronary Artery Obstruction Without Depressed Left Ventricular Function. Cardiol. Res..

[54] Zipes D.P., Wellens H.J. (1998). Sudden cardiac death. Circulation.

[55] Savard P., Rouleau J.L., Ferguson J., Poitras N., Morel P., Davies R.F., Stewart D.J., Talajic M., Gardner M., Dupuis R. (1997). Risk stratification after myocardial infarction using signal-averaged electrocardiographic criteria adjusted for sex, age, and myocardial infarction location. Circulation.

[56] Neri M., Riezzo I., Pascale N., Pomara C., Turillazzi E. (2017). Ischemia/Reperfusion Injury Following Acute Myocardial Infarction: A Critical Issue for Clinicians and Forensic Pathologists. Mediat. Inflamm..

[57] Kligfield P., Gettes L.S., Bailey J.J., Childers R., Deal B.J., Hancock E.W., van Herpen G., Kors J.A., Macfarlane P., Mirvis D.M. (2007). Recommendations for the standardization and interpretation of the electrocardiogram: Part I: The electrocardiogram and its technology: A scientific statement from the American Heart Association Electrocardiography and Arrhythmias Committee, Council on Clinical Cardiology; the American College of Cardiology Foundation; and the Heart Rhythm Society: Endorsed by the International Society for Computerized Electrocardiology. Circulation.

[58] Khalil H. (2022). Traditional and novel diagnostic biomarkers for acute myocardial infarction. Egypt. J. Intern. Med..

[59] Apple F.S., Collinson P.O. (2012). Analytical characteristics of high-sensitivity cardiac troponin assays. Clin. Chem..

[60] Maisel A.S., Krishnaswamy P., Nowak R.M., McCord J., Hollander J.E., Duc P., Omland T., Storrow A.B., Abraham W.T., Wu A.H. (2002). Rapid measurement of B-type natriuretic peptide in the emergency diagnosis of heart failure. N. Engl. J. Med..

[61] Nori H., Caruana R., Bu Z., Shen J.H., Kulkarni J. Accuracy, Interpretability, and Differential Privacy via Explainable Boosting. Proceedings of the 38th International Conference on Machine Learning, Proceedings of Machine Learning Research.

[62] Zhang Y., Cao Y., Xin Y., Liu Y. (2024). Significance of detecting cardiac troponin I and creatine kinase MB in critically Ill children without primary cardiac illness. Front. Pediatr..

[63] de Winter R.J., Koster R.W., Sturk A., Sanders G.T. (1995). Value of myoglobin, troponin T, and CK-MBmass in ruling out an acute myocardial infarction in the emergency room. Circulation.

[64] Robinson D.J., Christenson R.H. (1999). Creatine kinase and its CK-MB isoenzyme: The conventional marker for the diagnosis of acute myocardial infarction. J. Emerg. Med..

[65] Doudesis D., Lee K.K., Boeddinghaus J., Bularga A., Ferry A.V., Tuck C., Lowry M.T.H., Lopez-Ayala P., Nestelberger T., Koechlin L. (2023). Machine learning for diagnosis of myocardial infarction using cardiac troponin concentrations. Nat. Med..

[66] Dolci A., Panteghini M. (2006). The exciting story of cardiac biomarkers: From retrospective detection to gold diagnostic standard for acute myocardial infarction and more. Clin. Chim. Acta.

